# Rare Lung Malignancy Mimic: A Case of Daptomycin-Induced Acute Eosinophilic Pneumonia

**DOI:** 10.7759/cureus.56106

**Published:** 2024-03-13

**Authors:** Rebecca A Bowie, Christine Q Nguyen, LaRae L Seemann, Adrianna D Clapp

**Affiliations:** 1 Family Medicine, Mayo Clinic, Jacksonville, USA

**Keywords:** pleuritic chest pain, cough, dyspnea, lung cancer, daptomycin-induced acute eosinophilic pneumonia, daptomycin, acute eosinophilic pneumonia

## Abstract

Daptomycin is an antibiotic used for resistant Gram-positive organisms and has the rare side effect of inducing acute eosinophilic pneumonia (AEP). This condition can be fatal due to respiratory failure if not treated, as eosinophils migrate to the lungs and inflammatory cascades cause epithelial injury. Daptomycin-induced AEP can be misdiagnosed as bacterial pneumonia or malignancy, which may lead to unnecessary testing or treatments. Diagnostic criteria include dyspnea, fever, recent daptomycin exposure, infiltrates on imaging, eosinophils on bronchoalveolar lavage or peripheral eosinophilia, and clinical improvement with medication discontinuation. We present a unique case of daptomycin-induced eosinophilic pneumonia in a 72-year-old male with the chief complaint of dyspnea and initial concerns for lung cancer after a spiculated nodule was seen on imaging. Prior to undergoing a lung biopsy, repeat imaging showed a decrease in the suspicious nodule, reducing the likelihood of malignancy and prompting a re-evaluation of the history of the present illness and medication list. Daptomycin was stopped, and the patient’s symptoms and imaging improved. This case illustrates the importance of early recognition and appropriate treatment of AEP, which allows for complete clinical recovery.

## Introduction

Acute eosinophilic pneumonia (AEP) is a rare condition that occurs when eosinophils accumulate in the lungs and cause severe respiratory symptoms, which can be fatal [1]. It has been associated with smoking, inhalation exposure (occupational or recreational), medications (most commonly antibiotics or nonsteroidal anti-inflammatory drugs), infections, and idiopathic causes [1,2]. Daptomycin is an antibiotic known to cause AEP and is documented as an adverse reaction by the United States Food and Drug Administration (US FDA); this antibiotic is often used for resistant Gram-positive organisms, including methicillin-resistant *Staphylococcus aureus* (MRSA) and vancomycin-resistant *Enterococcus* (VRE) [1,3]. A 2012 report revealed 7 definite, 13 probable, and 38 possible cases of daptomycin-induced eosinophilic pneumonia [1], which highlights the rarity of this condition. A review of case reports showed that AEP from daptomycin had a median onset time of three weeks, often in patients undergoing treatment for osteoarticular infections, elderly patients ≥60 years old, and male predominance [1,4]. AEP is often associated with an increase in eosinophils on blood work and radiographic findings of pulmonary infiltration, ground-glass opacities, and consolidation on imaging, which can lead to lung cancer and metastatic disease as a possible diagnosis [4]. Daptomycin-induced AEP is an uncommon sequela of daptomycin therapy and can rarely result in chronic lung damage, steroid dependence, and death [5,6]. In this case, we present a 72-year-old male with pleuritic chest pain whose primary differential diagnosis was lung cancer; he was ultimately diagnosed with daptomycin-induced acute eosinophilic pneumonia.

## Case presentation

A 72-year-old male with active left lower extremity cellulitis on ceftazidime and daptomycin presented to the emergency department (ED) with pleuritic chest pain in October. He has a past medical history of restless legs syndrome, morbid obesity, sleep apnea, peripheral neuropathy, coronary artery disease due to a congenital split of the left anterior descending artery status post percutaneous transluminal coronary angioplasty with a stent, hyperlipidemia, prior L5-S1 spinal fusion, left knee arthroplasty, bilateral hip arthroplasties, and bilateral shoulder arthroplasty. Of note, the patient developed cellulitis in July with a recurrence in August and re-started antibiotics. In September, the patient had worsening cellulitis symptoms and was placed on daptomycin, dicloxacillin, and vancomycin. During his course, he contracted a peripherally inserted central catheter (PICC) line infection, and his antibiotic regimen was changed to daptomycin and ceftazidime.

A few weeks later, the patient was in the ED describing new chest pain as left-sided, non-radiating pressure, exertional and pleuritic in nature, associated with shortness of breath. On arrival, he was tachypneic with a respiratory rate of 36 but afebrile and normotensive. An electrocardiogram showed normal sinus rhythm with a right bundle branch block. Troponins and a comprehensive metabolic panel were normal. A complete blood count showed eosinophilia at 7.0% and absolute eosinophil count elevated at 0.64x10^9^/L (Table 1).

**Table 1:** Patient’s complete blood count results on presentation to the emergency department MCV: mean corpuscular volume; MCH: mean corpuscular hemoglobin; MCHC: mean corpuscular hemoglobin concentration; RDW CV: red blood cell distribution width, corpuscular volume; RDW SD: red blood cell distribution width, standard deviation; L: liter, g/dL: gram per deciliter; fL: femtoliter, pg: picogram

Component	Lab Value	Reference Range & Units
Leukocytes	9.1	3.4 - 9.6 x10(9)/L
Erythrocytes	4.49	4.35 - 5.65 x10(12)/L
Hemoglobin	12.8 (Low)	13.2 - 16.6 g/dL
Hematocrit	39.3	38.3 - 48.6 %
MCV	87.5	78.2 - 97.9 fL
MCH	28.5	25.4 - 32.7 pg
MCHC	32.6	32.1 - 35.6 g/dL
RDW CV	14.3	11.8 - 14.5 %
RDW SD	45.9 (High)	35.1 - 43.9 fL
Platelet Count	152	135 - 317 x10(9)/L
Mean Platelet Volume	9.9	7.6 - 10.8 fL
Neutrophils %	74.2	50.0 - 75.0 %
Immature Granulocytes %	0.3	0.0 - 3.0 %
Lymphocytes %	10.5 (Low)	18.0 - 42.0 %
Monocytes %	7.7	2.0 - 11.0 %
Eosinophils %	7.0 (High)	1.0 - 3.0 %
Basophils %	0.3	0.0 - 2.0 %
Neutrophils	6.74 (High)	1.56 - 6.45 x10(9)/L
Lymphocytes	0.96	0.95 - 3.07 x10(9)/L
Monocytes	0.70	0.26 - 0.81 x10(9)/L
Eosinophils	0.64 (High)	0.03 - 0.48 x10(9)/L
Basophils	0.03	0.01 - 0.08 x10(9)/L

**Figure 1:**
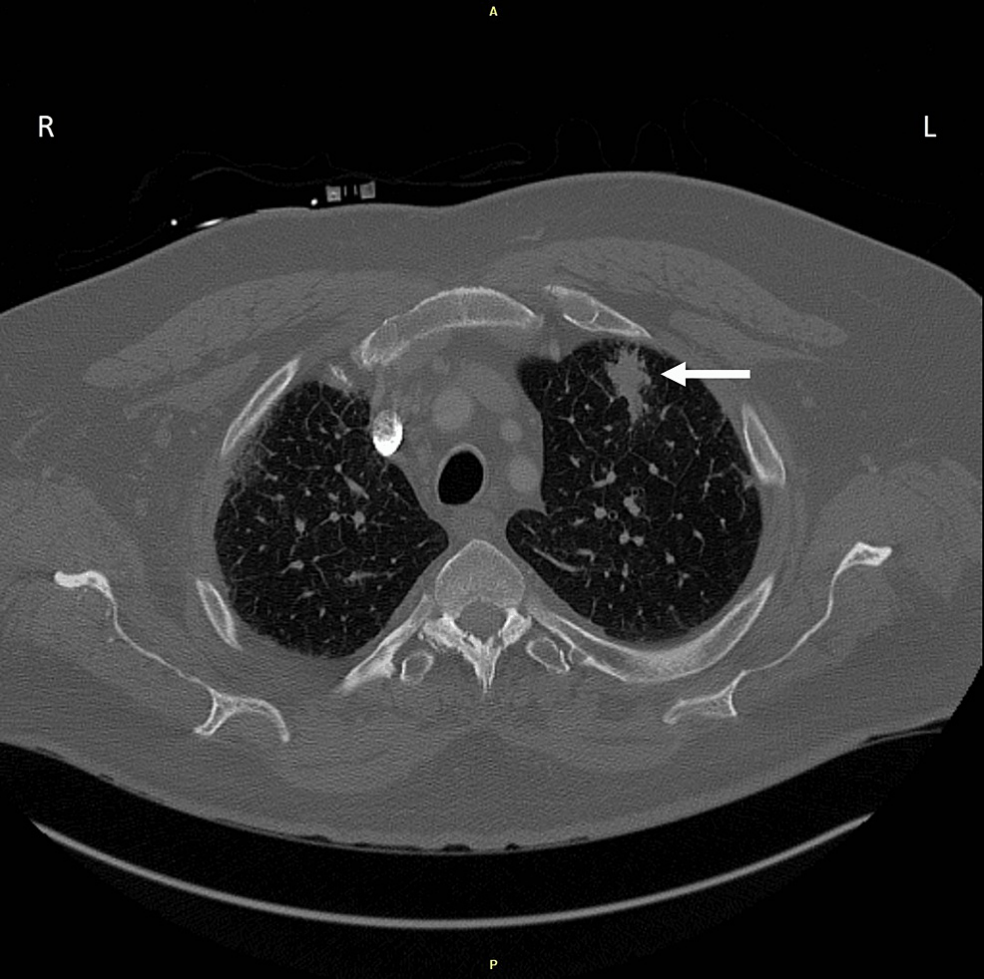
CT chest angiogram with IV contrast on initial presentation demonstrating an 18 x 26 mm solid nodule in the upper left lobe with irregular spiculated margins with concerns for malignancy (arrow) CT: computer topography; IV: intravenous; A: anterior; P: posterior; R: right; L: left

A computed tomography (CT) angiogram was obtained for concern of pulmonary embolism and showed a 26 mm spiculated nodule in the left upper lobe with adjacent irregular pleural thickening (Figure 1). It also showed multiple bilateral nodules and bilateral mediastinal and hilar lymphadenopathy, which was suspicious for malignancy. The patient was offered hospital admission, but he requested continued workup in the outpatient setting.

He was seen by his primary care team the following day. A CT-guided lung biopsy, brain magnetic resonance imaging (MRI), and positron emission tomography (PET) scan were arranged to investigate primary and metastatic lesions. An oncology appointment was also requested in preparation for the pending image and biopsy results.

**Figure 2:**
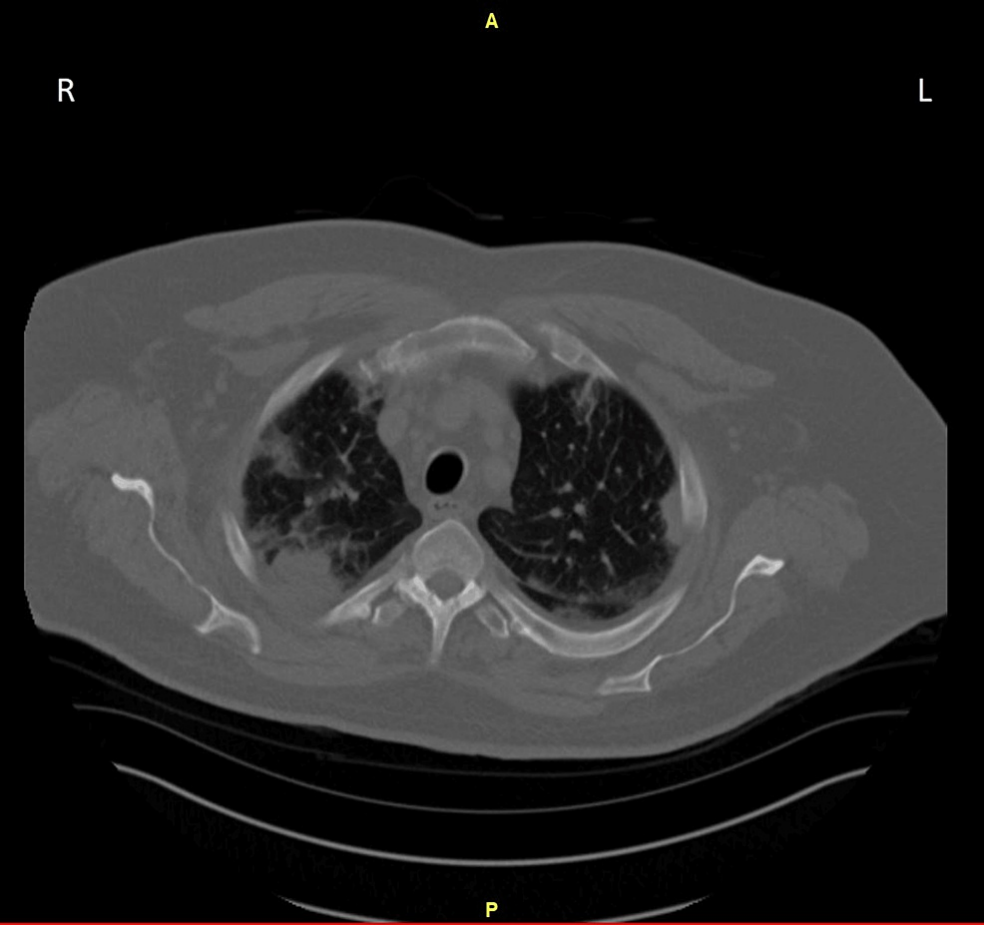
Follow-up CT chest without IV contrast five days after initial presentation, demonstrating a mild decrease in the size of previously seen peripheral nodular consolidation in the left upper lobe but interval worsening of similar peripheral wedge-shaped areas of nodular consolidations scattered in both lungs, predominantly in upper lobes These findings were worrisome for organizing pneumonia, eosinophilic pneumonia, or atypical infection. CT: computer topography; IV: intravenous; A: anterior; P: posterior; R: right; L: left

Five days after the initial presentation, the MRI brain was negative for lesions suspicious of metastasis. Repeat imaging in the interventional radiology suite prior to image-guided lung biopsy showed that his chest imaging had changed significantly, making malignancy less likely. The new findings were more suspicious for an evolving infectious process; therefore, the scheduled biopsy was aborted (Figure 2). He continued to have some left-sided chest pain and a mild increase in sputum production.

These updated images prompted a re-evaluation of his history. He had a follow-up with the primary care team (nine days after the initial ED visit) and was found to have a resting oxygen saturation (SpO_2_) of 91% and desaturated to 88% with exertion. He revealed an injury to the great toe on the plantar aspect while fishing, which progressed to infection and multiple trials of antibiotics managed by an outside infectious disease specialist. The patient denied fever, chills, or weight loss. He also denied recent travel or surgeries. He was a non-smoker. His medications were reviewed, and there was concern that daptomycin could be the culprit. He was on IV daptomycin at 6 mg/kg daily for 28 days prior to ED presentation. Additional testing, including sputum cultures, *Streptococcus pneumoniae* urine antigen, *Legionella* urine antigen, *Histoplasma* antibody, mycobacterial culture, acid-fast smear for *Mycobacterium*, *Blastomyces* antibody, and QuantiFERON gold, were negative.

The patient’s infectious disease doctor was notified of his clinical picture and agreed to stop daptomycin therapy due to concern for daptomycin-induced AEP, given changing infiltrates, hypoxia, and eosinophilia in the setting of one-month IV daptomycin. He was started on doxycycline as an alternative. A discussion was had with the patient about starting oxygen, but the patient politely declined as he was planning on minimizing exertion and had a follow-up with pulmonology.

At his pulmonology appointment three days later, he was prescribed albuterol for symptom control, and a repeat CT was ordered for three months. His three-month CT chest showed complete clearing of pulmonary parenchyma, including an absence of the pleural effusions that were seen on the initial CT scan.

## Discussion

Pneumonia can have a wide variety of presentations and etiologies. When pneumonia is suspected to be bacterial in nature, antibiotics can be the mainstay of treatment. Providers rarely entertain antibiotics as the cause of pneumonia. In this case, our patient presented with pleuritic chest pain, cough, and dyspnea clinically concerning for pneumonia. Imaging, in this case, supported a diagnosis of possible malignancy. With further investigation, a connection was made that the patient was started on intravenous daptomycin almost four weeks prior to presentation for ongoing cellulitis, supporting a diagnosis of daptomycin-induced AEP. With the discontinuation of the offending agent, the patient had improvement in symptoms and imaging, further supporting the presumed diagnosis.

The proposed pathophysiology of daptomycin-induced AEP involves macrophages presenting the drug or drug-hapten complex to a T-helper cell, which triggers the release of interleukin-5 (IL-5) [7,8]. Macrophages also release a chemokine known as eotaxin. Both interleukin 5 (IL-5) and eotaxin are thought to cause eosinophil migration to the lungs, and this inflammatory cascade results in acute epithelial injury [7].

Daptomycin-induced AEP can be diagnosed based on clinical history, laboratory results, and radiographic findings [1,9]. The following criteria have been used to diagnose AEP in different combinations to indicate definite, probable, possible, or unlikely diagnoses: 1) Exposure to daptomycin, 2) Fever, 3) Dyspnea, with increased oxygen requirement or requiring mechanical ventilation, 4) New infiltrates on chest X-ray or CT scan, 5) Bronchoalveolar lavage (BAL) with > 25% eosinophils or peripheral eosinophilia, and 6) Clinical improvement following daptomycin withdrawal [1,7]. A definite diagnosis was defined as having all six criteria, a probable diagnosis was defined as having five criteria (excluding fever), and a possible diagnosis was defined as having three criteria (excluding fever, dyspnea with increased oxygen, BAL, or peripheral eosinophilia) [1]. If a case does not meet these specific criteria, it would be considered an unlikely diagnosis [1].

A systematic review analyzed case reports and found the most likely findings were dyspnea (94%), fever (57%), peripheral eosinophilia (77%), and infiltrates/opacities on imaging (86%) [1]. In 13 of the 35 cases (37%), the infiltrates were bilateral. Other laboratory makers associated with daptomycin-induced AEP may include leukocytosis and elevated markers of inflammation like erythrocyte sedimentation rate or c-reactive protein [1].

Most patients respond well to the discontinuation of daptomycin with improvement within one to seven days [1]. An additional treatment option is systemic corticosteroids; this was prescribed in 66% of the systemic review's case reports [1].

The patient in our case report met five of six criteria for daptomycin-induced AEP, making it a probable diagnosis, including a 28-day exposure to IV daptomycin, hypoxia and dyspnea on exertion, peripheral eosinophilia, new infiltrates on chest CT, and clinical improvement with withdrawal of daptomycin. He had tachypnea in the ED and resting SpO_2_ of 91% in the clinic with exertional desaturation to 88%, meeting the criteria for oxygen. Four days after withdrawal of daptomycin, resting SpO_2_ was 97%. There was no bronchoalveolar lavage performed during the workup, but the complete blood count was notable for eosinophilia, which further supports the diagnosis. 

Our patient's initial chest CT showed a solid unilateral nodule with irregular spiculated margins, which caused suspicion of malignancy. There are no documented case reports where lung cancer was the primary diagnosis suspected. The unique aspect of this case was that the initial clinical presentation was so convincing of malignancy that he began an extensive workup, including a brain MRI, and had a scheduled lung biopsy, PET scan, and oncology consult.

The presentation of AEP in general has a wide range of differential diagnoses, including eosinophilic granulomatosis with polyangiitis (formerly Churg-Strauss syndrome), Goodpasture’s syndrome, fungal, parasitic, or bacterial pneumonia. Malignancy is often very low on the differential [8,10].

## Conclusions

Daptomycin-induced AEP is a rare adverse side effect of daptomycin use. The presentation of this complication can present non-specifically but can also mimic multifocal pneumonia and, in our case, malignancy. Clinicians should have a high suspicion for daptomycin-induced AEP when a patient on daptomycin therapy presents with new-onset lung pathology and nonspecific respiratory symptoms. Early recognition is important not only to prevent unnecessary testing in patients but also to stop the offending drug immediately so patient symptoms do not progress while workups are being conducted.
